# Spiritual Care: Motivations and Experiences through the Lenses and Voices of a Cohort of Spiritual Care Workers at an Established Hospice in Cape Town, South Africa

**DOI:** 10.1007/s10943-021-01232-7

**Published:** 2021-03-23

**Authors:** Ronita Mahilall, Leslie Swartz

**Affiliations:** Department of Psychology, Stellenbosch University, Private Bag X1, Matieland, 7700 South Africa

**Keywords:** Spiritual care, Palliative care, Hospice, South Africa

## Abstract

While palliative care is beginning to gain prominence in South Africa, spiritual care remains less understood. Spiritual care is less prioritised and, consequently, this service, if offered, is mostly entrusted to volunteers. It therefore becomes prudent to understand who these volunteers are, what motivates them to volunteer, and how they see spiritual care being sustainable in the future. A cohort of spiritual care workers from a prominent hospice in Cape Town, South Africa, participated in this qualitative study. The participants made suggestions about formalising spiritual care as well as making a call for a basic entry requirement into spiritual care work.

## Introduction

Palliative care in South Africa (SA) has, in relative terms, only recently been formalised as a discipline (Republic of South Africa, 2017). Both locally (Bhagwan, 2017; Chandramohan & Bhagwan, 2015; Ratshikana-Moloko et al., 2020) and internationally (Abel et al., 2013; Koper et al., 2019; Paal et al., 2015; Puchalski et al., 2019) there is consensus that spiritual care should play a pivotal role in palliative care.

There are many debates regarding how to classify countries. The “Global North” generally corresponds to countries categorised by the World Bank Atlas (World Bank Blogs, 2020) as high-income countries (HICs)—so, for example, Australia, which is a high-income country, is commonly viewed as part of the Global North, despite it being in the southern hemisphere. Countries in the “Global South” generally correspond to the World Bank low- and middle-income countries (LMIC) (World Bank Blogs, 2020). South Africa is a middle-income country. The distinction here is based largely on financial resources, though of course other factors are associated with relative wealth. In the Global North, where there are more resources, spiritual care workers are commonly health care professionals such as trained nurses (Balboni et al., 2007), medical doctors (Mati, 2016; Yang et al., 2017), and social workers (Callahan, 2015), or people with graduate qualifications in religion (Flannelly et al., 2003), but the picture is different elsewhere. In the Islamic world, for example, there is not a great deal of systematic research on spiritual care, despite the importance of spirituality as part of the Islamic faith (Asadi-Lari et al., 2008). In Africa, palliative care protocols tend to focus on more tangible aspects of palliative care such as medications and pain relief (Merriman & Harding, 2010). In SA, where the current study is situated, spiritual care is slowly being incorporated into the training programmes for nurses (Chandramohan & Bhagwan, 2015), social workers (Bhagwan, 2017), trained traditional healers (Campbell & Amin, 2014), and professional multidisciplinary teams (O’Brien et al., 2019). Despite this, we found from a national hospice survey that spiritual care is generally not prioritised at hospices, with the exception of hospices which are better resourced than others (Mahilall & Swartz, under review). Even in these better resourced South African hospices, though, unlike in the Global North, spiritual care services are often provided by volunteers rather than paid staff. These volunteers come from a range of backgrounds and may or may not have professional qualifications related to the provision of care.

There is a substantive body of work that explores volunteering within health care settings in SA, such as in hospitals (Lourens & Daniels-Felix, 2017), in primary health care clinics (Isaac et al., 2016; Johnson et al., 2020), community-based health care facilities (Swartz & Colvin, 2015), and home-based health care settings (Akintola, 2011; Morton et al., 2018). Much scholarly work talks about volunteers undertaking health care work in impoverished communities in SA (Akintola & Chikoko, 2016; De Wet, 2012; Nxumalo et al., 2016). Many of the volunteers themselves come from lower socio-economic ranks of society (Akintola, 2011; Barnard & Furtak, 2020). Care volunteers largely volunteer out of a desire to “give back” to their communities (Akintola, 2011) or extrinsically, in the hope of receiving a stipend (Morton et al., 2018). By contrast, spiritual care volunteering in hospice settings in SA, according to the limited data we have (Mahilall & Swartz, under review), seems more likely to be undertaken by older volunteers (some of them retired from formal employment) from higher socio-economic sectors, not with the expectation of receiving a stipend or moving into formal employment (Mahilall& Swartz, under review).

Therefore, there is a dependency and reliance on volunteers at quite a high skills level to offer spiritual care within a broader bouquet of hospice services (Mahilall & Swartz, 2021). It therefore becomes critically important to understand who volunteers as a spiritual care worker, what draws them to their work and sustains them in it, and what they believe is needed to sustain spiritual care into the future. With these questions in mind, we spoke to spiritual care providers at a hospice in Cape Town, SA.

## Method

St Luke’s Combined Hospices (SLCH), a prominent hospice in Cape Town, SA, offers a comprehensive palliative care service across a range of physical locations such as in-patient units, community-based day hospices where people who are terminally ill but not actively dying can congregate and receive care during the day, and in patients’ homes. Spiritual care services at SLCH are offered largely by volunteers.

SLCH currently has 23 registered spiritual care workers, of whom 15 are actively providing spiritual care services. The eight inactive spiritual care workers cited advanced age and personal illness as the main reasons why they have significantly limited their work at SLCH, resulting in their inactive status. We approached all active spiritual care workers to participate in our study, which forms part of a larger investigation into spiritual care issues in palliative care in SA.

Ethical approval for the study was obtained from the Stellenbosch University (10,237), Hospice Palliative Care Association (HPCA) (02/19), and SLCH itself. HPCA is a national association which has 104 member organisations affiliated to it (Drenth et al., 2018), with SLCH as one of those member organisations. Nine active spiritual care workers volunteered and gave written consent to be part of this study. The other six active spiritual care workers did not offer a reason for non-participation.

This was a qualitative study utilising an in-depth interviewing methodology which allowed for the capturing of rich, descriptive data (Hollway & Jefferson, 1997). One-on-one interviews were conducted which were approximately 90 min in duration. Prior to setting up the interviews, the participants gave both written and verbal consent. Ahead of the interviews the participants were given an Information Leaflet which highlighted, amongst other important information, confidentiality, protection of the data, and freedom to withdraw from the interviewing process or raise alternative views and arguments. As this is a sensitive topic, provision was made for psychological support for the participants at no cost to them. The interviews and subsequent three rounds of focus group discussions (FGDs) of approximately 120 min each were guided by a set of semi-structured questions (Appendix 1). Table 1 provides biographical data on the nine participants. Face-to-face interviews and FGDs were not possible due to lockdown regulations considering the COVID-19 pandemic. Consequently, interviews were conducted via virtual means such as Zoom and Skype, with the consent of the participants. As both authors are familiar with SLCH (one author works at SLCH and the other author conducted other research at the hospice), a co-facilitator knowledgeable in qualitative research was engaged to minimise researcher biase. The participants were informed and consented to this arrangement. The co-facilitator signed a pledge of confidentiality. The co-facilitator also facilitated the FGDs supported by the authors. The data obtained from the interviews and FGDs were recorded and transcribed. Through thematic analysis, a qualitative research method defined by Braun and Clarke as “identifying, analyzing and reporting patterns within data” (2006, p. 79), the data were coded and reviewed for emerging themes and subthemes. The themes and subthemes were then defined and named and analysed thematically.

**Table 1:** Biographical information on research participants

Participant number and “race”^a^ (B—black; C—coloured; W—white)	Gender (M—male; F—female)	Qualifications	Years of practice and experience as a spiritual care worker	Years of practice and experience as a spiritual care worker at SLCH	Religion	Languages spoken
1 (W)	F	BCom Honours (Economics)	3 years	3 years	Christian by birth. Currently: none	English, Afrikaans
2 (C)	F	Higher Dip. Education; Dip. Special Ed (Remedial); MPsych (clinical)	2 years	2 years	Islam by birth. Currently: none	English Afrikaans
3 (W)	F	Certified Lifeline Counsellor	30 years	28 years	Christian by birth. Currently: none	English
4 (B)	M	Grade 12. Lay counsellor in Church	25 years	7 years	Christian	English, Afrikaans, Xhosa, Zulu
5 (W)	F	BA (Social Work), Certificate: family constellation, Certificate: gender reconciliation, capacitar (Energy healing)	35 years	10 years	Quaker (Historical Christian denomination)	English, Afrikaans
6 (C)	M	BSc (Psychology), BA (Psychology)	25 years	15 years	Islam by birth but embraces all religions	English, Afrikaans
7 (B)	M	MA (Practical Theology), MA (Missiology)	15 years	13 years	Christian	English, Afrikaans, Xhosa
8 (C)	M	Dip in Theology, BA (Hons)	35 years	3 years	Christian by birth but embraces all religions	English, Afrikaans
9 (W)	M	Dip. Interior Design, Dip. Interior Architecture	6 years	6 years	Christian by birth. Currently: none	English, German, French

^a^In South Africa, as elsewhere, the use of “racial” terminology is complex and contested, and certainly still a source of debate and great pain. We make no claim for the scientific validity of the different “racial” categories, but the labels used still have social significance (Volmink et al., 2020). In the South African context, the term “Coloured”, which may well be the most contested, is an official term still used in government documents, refers to a very diverse group of people of mixed and diverse origin, with Afrikaans being the predominant language spoken (Volmink  et al., 2020). By contrast, the term “Black African” commonly refers to people who speak indigenous languages such as isiXhosa, spoken in the Western Cape. Under apartheid, the Western Cape was viewed as a preferential area in which “Coloured” people could live and work, whereas “Black Africans” were viewed as not being citizens of South Africa, but of racially defined “homelands”

## Results

Nine participants from SLCH, registered as active spiritual care workers at the organisation, volunteered to be part of this study. Through one-on-one interviews and FGD the participants shared their experiences as spiritual care workers providing spiritual care services to terminally ill patients in the larger prescient of Cape Town, SA. Three key themes emerged which we discuss in turn:

1. Becoming a spiritual care worker;
2. Being a spiritual care worker; and
3. Sustaining spiritual care services into the future.

### Becoming a Spiritual Care Worker

#### Varied Paths into Spiritual Care

The nine participants had varied and diverse entry points into spiritual care work, varied backgrounds, and a wide range of formal qualifications. Two participants were religious leaders such as pastors, as explained by Participant 7:

…well, let me say I’m a priest by calling. And now I started working here at St. Luke’s hospice as a volunteer, visiting people and doing sort of counselling and listening… (Participant 7)

Another participant began working in palliative care as a volunteer due to her personal interests in spirituality. Participant 2 shared her entry into spiritual care:

… I called SLCH’s Athlone [name of a community in Cape Town] Day-Hospice and said can I come and observe… So I went along that day…at the end of that the spiritual care coordinator invited me along to spiritual care. Initially it was just attending meetings and then when there was a gap in the ward they asked me to actually start visiting the ward as well. (Participant 2)

Participant 3 became a spiritual care worker by chance. Participant 3 explained:

…it evolved for me…I went in as a caregiver, I did training in both caregiving and lay counselling… I then joined Lifeline [a volunteer-based telephone counselling service similar to the Samaritans in the United Kingdom] and…the spiritual thing just evolved somehow… (Participant 3)

His personal interest and experiences motivated Participant 6 to volunteer at the hospice in another capacity, as an administration volunteer, but he later transferred into spiritual care work. Participant 6 explained:

…needing a volunteer to come and assist with palliative care…to the terminally ill Muslim patients… They needed a male particularly, who was Muslim and who had an interfaith background. And that’s how I got started in spiritual care in 2008 at St. Luke’s hospice. (Participant 6)

Only one of the participants indicated that he began his spiritual care work through a formal job application for a vacant post. The varied entry points into spiritual care could have implications for how spiritual care is viewed within and outside the hospice. We unpack this for implications in greater detail later on.

#### The Role of Religion

Although some of the participants had specific religious affiliations at birth, such as Christianity, as participant 8 explained:

…as a Christian, in the Christian faith there is a scripture that says that God is the God who heals. Many a time we find people coming here with that same conviction. But the doctor says you are dying and they still live with that hope and conviction that they will be healed; but they die…it’s like God verse science… Personally I would say my idea of God has changed… I would not necessarily follow the route of exclusivity [as a Christian] where I did that in the past. (Participant 8)

and Islam, as Participant 2 explained:

…when you look at me I am Muslim, but I don’t wear a scarf and I’m really more spiritual than religious… My dad I saw as the embodiment of a particularly spiritual person. But he never went to the mosque, but he sat with prayer beads in his hands all his life. You know, so I grew up in a much more liberal kind of way from…and respect for everybody. (Participant 2)

participants tended to adopt a flexible and philosophical approach to religion and spirituality. The participants described their religious beliefs largely in spiritual and existential terms, and not in conventional religious dogma. As Participant 3 put it:

…Sunday School, Gentle Jesus, all those simplistic religious things; I know that there is something bigger than me. I know it! I know, I talk about God with patients on a daily basis. The word God doesn’t impact me. In fact, the spirituality assists in the religious side, it gives me more of an ‘in’ [into] spirituality. I didn’t find my spirituality through my belief in God. It’s the other way around. (Participant 3)

Participant 9 had this to say about his religiosity and that of the patients in his care:

It is appropriate to always consider a patient’s cultural, religious, social and or any other circumstances and situations as well as to be aware of mine. However, I found in my, by now seven years of practical counselling of palliative patients who are approaching their end of life stage, that these differences appear to fade into the background, and a universal understanding between all humans emerges, as long as there is a movement in consciousness from an intellectual understanding of the prevalent conditions of the patient, to a more heartfelt approach to the patient’s suffering. (Participant 9)

There was consensus from all participants that to listen to patients with compassion and to be guided by the patient in their spiritual journey, as opposed to their own as spiritual care workers, and to go with the patient to wherever that conversation may lead to, was fundamental to a meaningful therapeutic relationship. Participant 1 captured this in the following way:

I’ve read so much, I’ve been on these courses [counselling courses]…but you need to obviously always look at the whole person, not just looking at someone’s religion or what you read in a book. You’re looking at their body, their emotions, their spirit—you’re looking at the whole of that person in front of you…really you’re led by the person…if a dying person wants to talk, they want to talk about something that’s in their head…you just listen with compassion and go where the patient leads you. (Participant 1)

### Being a Spiritual Care Worker

#### Finding Joy in the Work

The participants expressed satisfaction and fulfilment with the spiritual care work that they undertake and highlighted the positive benefit spiritual care services bring to the terminally ill patient and, as a result, to them as spiritual care workers, something we come back to in the discussion. Participant 2 explored the different ways in which patients can derive spiritual support, such as from outings, and using art and music to open discussions on existential issues. She shared this case study that brought her and the patient closer in the spiritual care session:

I asked the patients to give me the name of a song that had special relevance for them and then I got the songs on a play list for them… This particular uncle Tienie was an 85-year-old man who told us about how he came from a rural area and how he came to Cape Town. The story that he told around the song was absolutely amazing… So you actually get people talking to each other, really seeing each other and as I say there’s nothing like music to remind you of the past and when you were healthy and happy and all of that. So that is one of the big highlights and ways to get my patients talking and brings me such joy. (Participant 2)

#### An Individualised Approach

These observations draw attention to the highly individualised nature of spirituality, and that spiritual comfort can be obtained from a variety of activities, and not only from counselling. Some such activities are storytelling. Participant 8 highlighted the importance of storytelling which helped his patient resolve unfinished issues with the patient’s mother:

He said that he had grown up in school hostels all his life. So he was still bearing that resentment [against his mother for sending him to boarding school] and he opened up to me and he said that he needed to get to the point where he could consider forgiving his mother and reconciling with his mother who was at that time already 81 to 82 years old. Then I got his mother to come and see him a few days before he died and they had made peace and as I said he died a few days after that. (Participant 8)

#### Spiritual Care in Context

Participant 2 referred to the simple things, such as getting a favourite cool drink for a patient, as rewarding as a spiritual care worker:

I made a cake on her [patient] birthday which she had in the ward and all the nurses came to sing for her…she [the patient] hadn’t been eating for days and she felt like drinking Spar berry drink [cool drink] and I fetched it for her and I felt as close to being a death doula for her as one can in this South African context. (Participant 2)

For Participant 3, it was simply listening to patients tell stories about who they were when they were younger or healthier that brought satisfaction and inspiration to her as a spiritual care worker:

I had a lady, from another African country, who was dying of HIV and cancer. She was in a wheelchair. She was really exquisite, a really beautiful bone structure. We chatted for a while and she was extremely depressed. And I said to her, ‘tell me who you are, what were you before all of this?’ She said, ‘I was a dancer’. And the change in that woman was instant. And I said, ‘tell me about the dancing? What did dancing mean to you?’ She just spoke for an hour until she was exhausted…happy but exhausted. That has lived with me for a long time because she became animated. She became a human being. She didn’t become the HIV or the cancer. (Participant 3)

Participant 7 highlighted how he engaged with spiritual and existential issues outside of the conventional frameworks of religion and how he allowed for the patient to lead the session on the patient’s own terms and in ways that the patient found meaningful to them:

Another patient said to me, ‘You know this is the first time a person listens to me. Many people will come with advices saying do ABCD, and God is doing this and that. But a person who listens and I talk, I air out what is going on. What is within myself. You listen and after that you don’t even say can we pray? If I want prayer, I will say would you please Father pray?’ That also was a very good lesson for me. (Participant 7)

### Sustaining Spiritual Care Services into the Future

The participants had diverse academic and professional backgrounds (see Table 1) and expressed the need to professionalise spiritual care services, something we come back to later in the discussion. Participant 8 explained:

I would like to be trained or helped to understand family dynamics better. And then also because in many homes we meet the children of patients who are drug addicts so I would also like to become au fait with ways of helping those guys. Say maybe rehabilitation principles…and to understand the medical terms that won’t normally be understood by [lay] people. There are a lot of medical terms that are brandished about here in the discussions, especially in ward round and from time to time I have to ask, hey, just explain to me what you mean by that. And then psychology, the psychological side. And it would be good to let people know the laws surrounding social work practice because many a time there is the idea of children who need to be taken care of after mom has died and maybe when it comes to Wills and a person need to decide what’s going to happen to his assets and the spiritual carer is often the person to whom they want to talk about it because they do not understand the legal side, so maybe some of the training that social workers would get in the laws regarding Wills and adoptions, and a number of other things, can be given to us as well. So yes, you heard right, we must professionalise spiritual care work. (Participant 8)

Participants recognised that people can become involved in spiritual care work through a variety of different career paths. Participant 6 suggested a qualification that could be an ideal basic academic foundation for spiritual care workers, something we come back to in the discussion:

I think that…you would need to have…some knowledge of psychology…you would need to understand human behaviour…you need to understand emotions…you need to understand why people or some people are angry, or resentful, in a sense of depression, what to do, how to handle depression and anxiety, and familiarise yourself…so I think a course or two or three in psychology or sociology, and social welfare work and so on would be necessary. …but they should have some accreditation or diplomas to that affect. Otherwise, you know it could go haywire. (Participant 6).

Alongside finding an equitable balance between professionalising and standardising spiritual care services, the participants felt strongly that it was critical to achieve this without making spiritual care services so rigid that more informal creative approaches such as using art and music may then be excluded. Participant 3 and Participant 5 stated the following:

We can professionalise it (spiritual care services), but we would still have to have trained people like myself or our team that could work and who did not have the (academic) degree. I wouldn’t like to put it (spiritual care) in a box again. (Participant 3).

I have brought poetry onto the ward if it is appropriate and I remember there was a woman who told me that Tina Schouw was her favourite singer and I brought a CD and she played it when we had a CD recorder and she wanted that at her funeral. We can work on the creativity, I won’t go into the whole writing letters and making cards, and images. There was a woman who was reported to be a Buddhist and we knew very little about her but she had her iPad and she was looking at what Buddhists do and right back to her childhood, she was looking back at pictures and through her failed marriage and into Buddhism and she shared that with me. What have people got next to their beds, what are their books, what are their things that they have next to their beds. We can pick up and enlarge a part of them that they are presenting in a creative way… We had an American woman, who came to St Luke’s with her students and there were a couple that were with someone who was dying and she gave them a cello concerto that was such a beautiful farewell with the husband and the wife. (Participant 5)

The participants further expressed that given the highly individualised approach to spiritual care services any standardisation and professionalisation that could take place should still allow for a practical skills development component, typically in the form of mentoring or an internship of some kind for face-to-face learning to take place. Participant 5 expressed the following (the “volunteer’s programme” referred to here by participant 5 is an orientation programme, sometimes referred to as a “screening” programme or “Basic Hospice Course”, offered to all prospective volunteers to SLCH that seeks to showcase the multiple avenues in which volunteer work can be undertake):

There is a basic need that if somebody has done the volunteer’s programme, you don’t just become a spiritual counsellor. I mean people do a volunteers programme and they hear one person speak for one session of spiritual care and they think death is quite sexy at the moment…we need experiential training for spiritual carers and we need mentoring, we need people that have had the experience to run a peer group or talk it through or they will fall through the gaps. So if you are working as a team, certainly in the ward [in-patient unit], the opportunity to discuss and to be mentored and co-counselled with somebody else that for me was a very big growing point. We used to have case discussion as well and go in [home visits] with the nurses too and that is where the growing is. You should go through these process and then you should have a mentor and then you should…come onto the ward [after] having actually experienced the community-based day hospices, because you know some people might come from backgrounds where they’ve never had any contact with somebody who’s from Gugulethu or from Grassy Park [predominantly marginalised communities result from the apartheid regime]. (Participant 5).

## Discussion

We discuss the following three themes which emerged with prominence.

### Spiritual Care Practice

It became clear that the participants of this study were committed to the spiritual care services they provided and that they served as a synergistic satisfier (Max-Neef, 1987) in which the mutual benefit of the patient/carer relationship was symbiotic. The participants in this study negotiated entry into spiritual care services at SLCH through diverse touch points and for various personal reasons. This was expected, as it is well documented, locally, and internationally that volunteers volunteer for a myriad of personal reasons (Akintola, 2011; Daute et al., 2019; Mati, 2016; Morris et al., 2015; Tapp et al., 2019). We were struck by the seemingly diverse backgrounds and qualifications of the participants drawn to spiritual care work which intimated that there was no clear career path leading into spiritual care work and neither did it appear that there was a basic prerequisite qualification for entry into spiritual care work. Research abounds on the importance of spiritual care workers in palliative care, but this is largely premised on the presumption that spiritual care workers have a health care background (Best et al., 2020; Kestenbaum et al., 2017; Paal et al., 2018). This is not the case in the South African context.

### Credentialing Spiritual Care

In SA, spiritual care seems not to be ensconced in a core or formal curriculum, but rather it appears to be an ancillary course taught at some institutions of higher learning (Linda et al., 2015). Germane to this and emerging from a national study of training needs of spiritual care workers in hospice palliative care settings, a set of soft and hard skills were suggested as a key skills-set for spiritual care workers in SA (Mahilall & Swartz, under review). Similarly, in the northern hemisphere, there is increasing consensus on the importance of training for spiritual care workers in spiritual care (Isaac et al., 2016; Paal et al., 2018; Puchalski et al., 2019). Advocacy for postgraduate palliative care education for all health care workers in Europe has gained momentum by seeing the voices of the World Health Organization (WHO Global Observatory for eHealth, 2010) and European Association for Palliative Care (EAPC) joining the call. In SA, while still very new as a recognised health care service, palliative care is gaining momentum, as the National Department of Health (NDoH) has prioritised palliative care at the primary, intermediary, and tertiary health care levels (Republic of South Africa, 2017). Consequently, the NDoH has also prioritised the scaled-up training of cohorts of different health care professionals, community health workers and care volunteers in palliative care (Gwyther et al., 2018). This is a positive step in the right direction in establishing palliative care, and especially spiritual care, as it begins to gain some ground towards professionalising and standardising this important work by providing some guidelines for spiritual care practice.

The participants of this study felt that part of the process of sustaining spiritual care services at hospices would be to provide recognised training in spiritual care, to develop a spiritual care code of ethics, and to have spiritual care services registered with a professional body. Similar calls have been made by Linda et al. (2015) for formal education in spiritual care in nursing; Mthembu et al. (2016) for formal education in spiritual care in health sciences; Bhagwan (2017) for formal education in spiritual care for social workers; and Gwyther et al. (2018) for training for hospital-based professionals. Participants expressed the importance of screening potential spiritual care volunteers who are looking to enter into spiritual care work, not only to assess their skills and competencies, but also to assess their personal attributes and motivation for wanting to do spiritual care work; nuggets of suggestions which call for greater investigations and understanding and which could have value to SLCH and other hospices in SA in the recruitment of spiritual care volunteers, as well as positioning spiritual care within the palliative care framework at hospices in SA.

#### Spiritual Care as a Crisis Intervention

The current global pandemic has not only brought about a global financial crisis but has also forced health care systems to focus more acutely on end-of-life care (Baldwin-Ragaven, 2020). Communities and families have historically found comfort in each other in times of personal or national disasters through religion and philosophy (Mati, 2016). Roman et al. (2020) suggest a more contemporary view that spiritual care is part of the human psyche and consequently forms part of human care, as well as care for society, families, patients, and care providers. Studies have also shown that patients’ reliance on spiritual care increases during life-altering events (Mthembu et al., 2017), but while many health care professionals recognise the value of spiritual care interventions for their patients, many felt inadequate in providing that care themselves (Mthembu et al., 2016). While Roman et al. (2020) call for a collaboration of spiritual care practitioners to address the accelerated need for spiritual care during the COVID-19 pandemic, this pandemic has also shown that there is an immediate need to better equip existing health care professionals with skills and training in spiritual care as well as grow a volunteer force of adequately trained spiritual care workers, something the participants of this study called for as well.

## Limitations of this Study

While some important perspectives and views about spiritual care practice and understanding the motivations of spiritual care workers in doing this work were identified in this study, it is important to acknowledge the limitations of this study. The first limitation was that only spiritual care workers from one hospice, SLCH (albeit the oldest and largest hospice in the Western Cape, SA) were included in this study. The second limitation was that the methods for this research were limited to individual interviews and FGDs. An expansion of these methods, to include, for instance, observations or photo voice research methods, could have provided additional rich data in understanding the experiences of spiritual care workers providing care in hospice palliative care settings. Thirdly, whilst this study cannot be generalised in the statistical sense of the word, there was widespread representation (albeit purposive sampling of only spiritual care workers) across all communities serviced by SLCH, suggesting that these triangulated findings have some integrity.

## Conclusion

The importance of spiritual care services in palliative care is well documented both locally and internationally. While the literature in the Global North supports this assertion, it is imperative to bear in mind that the large body of such research is based on the premise that spiritual care workers have a prior recognised care background in, for example, medicine, psychology, or chaplaincy before entering the spiritual care services field, whereas the reality in the Global South, and in SA in particular, is that spiritual care work is taken up largely by volunteers with diverse backgrounds and educational qualifications. Amongst our participants, admittedly a small group, but insider experts, there emerged a felt need to grow spiritual care practitioners and to formalise spiritual care services in hospice palliative care settings in SA. The time is now ripe to explore these issues as palliative care negotiates its rightful place in the national continuum of health care system.

## Recommendations

This study suggests many directions for future research. Some suggestions for future research include:

### Entry Requirements

This study highlighted the diverse qualifications and entry paths into spiritual care work in the SA hospice context, which suggests that there does not exist a basic entry requirement into spiritual care work at hospices in SA. Is a basic entry requirement necessary? This study further suggests that according to people currently doing the work, this may well be necessary. We call for open dialogue and more empirical studies to be undertaken in other provinces in SA, as well as extended further to the Global South, with the aim of understanding and addressing entry criterion into spiritual care work.

### Further Research on Understanding the Motivations of Spiritual Care Workers

It may also be helpful to understand the motivations of prospective spiritual care workers to undertake spiritual care work at a hospice. Further open dialogue and research are needed on this topic, which may be helpful to hospices seeking to employ spiritual care workers.

### Satisfaction Assessments

It may also be useful to undertake satisfaction surveys and further empirical studies with practicing spiritual care workers with a view to using that data to scope spiritual care as a recognised discipline in palliative care.

## Data Availability

The datasets used and/or analysed during the current study are available from the corresponding author on reasonable request. Written informed consent was obtained from all individual participants included in the study.

## Appendix 1: Guiding Questions for Semi-Structured Interviews

**Table:**

Collapsed Interview Questions	Original Questions
A: How and why you became a spiritual care worker	1. Tell me about how you became a spiritual care worker?
	2. Tell me about why you became a spiritual care worker?
B: Positive highlights of spiritual care work	3. Share with me some of the highlights of your work in spiritual care?
	4. Can you cite some examples to illustrate those highlights?
C: Chat	
D: Difficulties experienced in spiritual care work	5. Share with me some of the difficulties of your work in spiritual care
	6. Can you cite some examples to illustrate those difficulties?
	7. How did you mitigate those difficulties and challenges?
E: Skills and training needed for SCW	8. What would you consider to be your training needs as a spiritual care worker?
	9. What would your thoughts be in professionalising spiritual care?
	10. How would you envisage this happening?
F: Describe your spiritual care work and spiritual care work in general	11. How would you describe your work as a spiritual care worker at SLCH?
	12. Could you perhaps share your experiences of how you engaged with patients as a spiritual care worker?
	13. What is your understanding of spiritual care?
G: Is there a distinction between religion and spirituality?	14. For you, is there a distinction between spirituality and religion?
H: What role does your religious faith play in your spiritual care work?	15. What role does your religious faith play, if any, in your delivery of spiritual care services?
I: What is the spiritual care framework you use?	16. What is the framework within which you provide spiritual care intervention?
J: IDT and spiritual care services	17. Can you describe your understanding of the IDT at SLCH?
	18. What role does spiritual care play within the IDT?
	19. How would you describe your work as a spiritual care worker within the IDT?
	20. How would you describe your work within the spiritual care team at SLCH?
	21. Do you feel that having spiritual care workers working at SLCH brings about positive change to the service the hospice offers?
	22. Can you cite examples to illustrate that?
	23. Do you feel that having spiritual care workers working in an IDT at SLCH brings about positive change to the approach of the IDT to the patient?
	24. Can you please cite examples to illustrate that?
K: Diversity and spiritual care	25. Given the range of diversity in SA how would you say this affects the way you provide spiritual care within your hospice?

*SCW* spiritual care workers, *SLCH* St Luke’s combined hospices, *IDT* inter-disciplinary team,*SA* South Africa
